# Beyond critical and courageous thinking: let’s make medical education better

**Published:** 2018-07-27

**Authors:** Marcel D’Eon

**Affiliations:** 1 University of Saskatchewan, Saskatchewan, Canada

Can we agree that making the world a better place is a valuable goal? If our world happens to be medical education, at least for a good part of the day, could we agree that making medical education just a little better than it was before is a priority? While I wrote for the last issue of the CMEJ (and I still believe) that we are in desperate need of critical and courageous thinking,^1^ we need to move beyond thinking to doing. And not just doing what’s easy and expedient^2^ but making the sacrifice (personal or otherwise) to choose the best way forward for our learners and their patients.

Our cover artwork “Overdosed” pleads for harm reduction strategies. Can we set aside our prejudices and teach each other how to make life better for people with addictions rather than heaping scorn and judgmental curses upon them? Instead of choosing the expedient (ignoring or punishing), could we meet them where their addiction has tossed them? Could we perhaps protect them from the perils of the pit into which they fell with a chance that they may actually one day ask us to help lift them out?

Faculty development, by nature and purpose, tries to make medical education better by influencing the teachers of today and tomorrow. Medicine itself (and other helping and/or health professions) have made a commitment to make the lives of patients better, if not to cure then to comfort and maybe both. Making our world better is a common and shared value. Surely, we can see this is what we need to do in our roles as medical educators.

The CMEJ too is part of this collective effort. In service of the Canadian and international medical education communities we have grown from just two issues and 75 pages a year in 2010 to four issues and 400 pages a year in 2016, 2017, and likely in 2018 as well. At the same time, we have been strengthening our processes: the length of time it takes us to get the first decision on a submission back to authors has plummeted from over 240 days to about 80 days and our quality is climbing (with an acceptance rate of about 15%). We are planning celebratory 10^th^ anniversary events for 2020, not because we have attained perfection and not because we are the best in the field, but because we are getting better (with leaps and bounds) and making medical education better because of our concerted efforts!

Over the years as the editor, I have faced difficult decisions. A few times, after one or more rounds of revisions and after giving the authors the impression that we would very likely publish their paper, I have discovered upon second or third reading, a fatal flaw (or two). Upholding the quality of the journal is one of my main responsibilities. Facing the disappointment and wrath of authors is my least favourite task. Bracing myself, I have on occasion explained that, sorry, we cannot publish the paper after all. Sometimes the authors have surprised me; with another honest read through a window I had opened up for them, they decided to make their paper better instead of clinging to their disappointment. In the early days of the new CMEJ I firmly (and somewhat fearfully) expressed my views about the direction I thought the journal needed to take. Having exercised some patience and faith, I believe we are definitely heading in the right direction. Those are two examples where I chose the more challenging path and rejected the expedient. In these two examples it seemed to work out for the better.

Sometimes we choose the expedient. When powerful basic science departments cling to traditional content and delivery methods it is easier to surrender than exercise deliberate and difficult leadership to make medical education better. That cutting edge of tradition^1^ usually wounds medical students, not faculty.

This talk of powerful, vested interests leads us to the articles in this issue and especially the essay by Jaworsky, “A settler physician perspective on indigenous health, truth, and reconciliation” along with the accompanying commentary by Smith. These are required reading for students, residents and practicing health professionals.

Klowak *et al.* in “Predictors of medical student interest and confidence in research medical school” wanted to determine students’ interest in research and their skill level using self-reports on a survey. Among other things they found that higher ratings of supervisors’ understanding of research were associated with greater interest in research (OR=2.08; 95% CI=1.27–3.41). Here’s a way to make medical education better!

Hodwitz and her team in “Redeveloping a workplace-based assessment program for physicians using Kane’s validity framework” explain the four inferences of Kane’s model (*Scoring, Generalization, Extrapolation*, and *Implications*). They believe that Kane’s framework was valuable for guiding the redevelopment process and for systematically collecting validity evidence. They state that discussions about validity are crucial for the development and evaluation of workplace based assessment programs.

Dallaire et al. in “Interdisciplinary teaching in family medicine teaching units: the residents’ points of view” acknowledge both that interdisciplinary teaching is the norm in Canadian family medicine residency programs but that little is known about family medicine residents’ own perspectives of it. Using a mixed methods design with content analysis they found that residents felt that interdisciplinary teaching works best when the teachers adapt to the specific needs of residents in family medicine.

We can all use these words of wisdom to make medical education better.

Coutin and colleagues in “Missed opportunities: are residents prepared to care for transgender patients? A study of family medicine, psychiatry, endocrinology, and urology residents” compared perceptions of trans-care education and training across these residency training programs at the University of Toronto. Using a survey, they found that only 17% of all participants predicted they would feel competent to provide specialty-specific trans-care by the end of their residency and only 12% felt that their training was adequate to care for this population. It seems that there is a lack of exposure and trans-related patients and relevant teaching.

Morgan *et al.* in ““They don’t have the history and the stature:” examining perceptions of Caribbean offshore medical schools held by Canadian medical education stakeholders” explore how these institutions are perceived by those in professional and decision-making positions at Canadian institutions where graduates intend to practice. They found that the 13 Canadian medical education stakeholders they interviewed believed that these medical schools are a) at the bottom of an international hierarchy, are b) heterogeneous in quality of education and student body; and c) have a unique business model, characterized by profit-generating and serving international students. They also learned that the core principles of social accountability in Canadian medical education are incompatible with the offshore medical school model practiced in the Caribbean.

Khalife and compatriots in “Transitioning towards senior medical resident: identification of the required competencies using consensus methodology” used a Delphi technique to poll recent graduates and practicing internal medicine physicians to identify the competencies when transitioning to a senior role within Internal Medicine training. They initially identified 83 competencies while 77 reached consensus after three rounds.

Content is one of two main components of any curriculum and more attention to the selection of core content can only make medical education better.

Docherty-Skippen and Beattie in “Duoethnography as a dialogic and collaborative form of curriculum inquiry for resident professionalism and self-care education” explore the transition to practice. Duoethnography invites *extrospection* (a self-examination through a coach), thoughtful observation and negotiated consideration. While guided by eight principles, the approach is not prescriptive. Its advantages include: 1) the capability to foster self-reflexive and transformative learning; 2) the versatility to accommodate learner diversity; and 3) an adaptability for use in different social, situational, and ethical contexts.

McConnell and her team in “Does testing enhance learning in continuing medical education?” did not find that it did, surprisingly. For the CME activities they tested, they found no testing effect on performance eight weeks later. This is an important contribution to the literature. We need to identify those factors that will contribute to a testing effect, or better yet, how to enhance learning.

Dahn and her team from Dalhousie in “Transition to practice: creation of a transitional rotation for radiation oncology” asked their multidisciplinary participants to rank learning objectives expected to be mastered by a graduating resident. They then calculated mean importance scores for each objective and used the results to guide the development of a transition to practice rotation.

Coderre *et al.* in “Are we failing to build on the scientific basis of medicine?” make an important point using two unusual and clever sources of data: a tally of publications by topic and themes chosen for the annual Canadian Conference on Medical Education. They question why the Canadian national medical education organizations have failed to introduce or promote changes that would compel or at least encourage Canadian medical schools to heed the recommendation from the Future of Medical Education in Canada report to “build on the scientific basis of medicine.” The evidence they have collected indicates that there has been little emphasis on the scientific basis of medicine since 2010. They offer some explanations.

Weston, in an accompanying commentary curiously and deliberately entitled “Do we pay enough attention to science in medical education?” argues persuasively for less, not more, biomedical science in an already overcrowded medical school curriculum. This has been a contentious issue for years.^3^ One wonders if we will choose the expedient rather than the meaningful. So far, we have been on the cutting edge of … tradition, with predictable results.

The researchers, authors, and artists published in this issue and others have all committed time and energy into making medical education better. We at the CMEJ earnestly hope that you will find these items inspiring and worthwhile. They help to point a way forward that may not be expedient but is meaningful and valuable. We are all aiming to make medical education better.
